# Towards progressive regulatory approaches for agricultural applications of animal biotechnology

**DOI:** 10.1007/s11248-021-00294-3

**Published:** 2022-01-09

**Authors:** Eric M. Hallerman, Justin P. Bredlau, Luiz Sergio A. Camargo, Maria Lucia Zaidan Dagli, Margaret Karembu, Godfrey Ngure, Rhodora Romero-Aldemita, Pedro Jesús Rocha-Salavarrieta, Mark Tizard, Mark Walton, Diane Wray-Cahen

**Affiliations:** 1 Virginia Polytechnic Institute and State University, Blacksburg, VA USA; 2 U.S. Department of Agriculture, Washington, DC USA; 3 EMBRAPA—Brazilian Agricultural Research Corporation, Juiz de Fora, MG Brazil; 4 School of Veterinary Medicine and Animal Science, University of Sao Paulo, São Paulo, SP Brazil; 5 International Service for the Acquisition of Agri-Biotech Applications AfriCenter, Nairobi, Kenya; 6 ISAAA SEAsia Center, Los Banos, Philippines; 7 InterAmerican Institute for Cooperation On Agriculture, San José, Costa Rica; 8 Commonwealth Scientific and Industrial Research Organisation, Geelong, VIC Australia; 9 AquaBounty, Maynard, MA USA

**Keywords:** Genome editing, Genetic modification, Genetic engineering, Public policy, Regulatory cooperation, International trade, Public acceptance, Livestock

## Abstract

Traditional breeding techniques, applied incrementally over thousands of years, have yielded huge benefits in the characteristics of agricultural animals. This is a result of significant, measurable changes to the genomes of those animal species and breeds. Genome editing techniques may now be applied to achieve targeted DNA sequence alterations, with the potential to affect traits of interest to production of agricultural animals in just one generation. New opportunities arise to improve characteristics difficult to achieve or not amenable to traditional breeding, including disease resistance, and traits that can improve animal welfare, reduce environmental impact, or mitigate impacts of climate change. Countries and supranational institutions are in the process of defining regulatory approaches for genome edited animals and can benefit from sharing approaches and experiences to institute progressive policies in which regulatory oversight is scaled to the particular level of risk involved. To facilitate information sharing and discussion on animal biotechnology, an international community of researchers, developers, breeders, regulators, and communicators recently held a series of seven virtual workshop sessions on applications of biotechnology for animal agriculture, food and environmental safety assessment, regulatory approaches, and market and consumer acceptance. In this report, we summarize the topics presented in the workshop sessions, as well as discussions coming out of the breakout sessions. This is framed within the context of past and recent scientific and regulatory developments. This is a pivotal moment for determination of regulatory approaches and establishment of trust across the innovation through-chain, from researchers, developers, regulators, breeders, farmers through to consumers.

## Introduction

A number of global trends contribute to the need for heightened investment in the development and distribution of genetically improved agricultural animals. This need is heightened by increased demand for animal-based protein due to growing prosperity and decreasing arable land for agriculture. Further, ongoing climate change is increasing physiological stress upon production animals, contributing to outbreaks of new diseases and expanding ranges of pests and disease vectors. There is also a growing interest in animal welfare among farmers and consumers. Against this background, the animal production sector needs to genetically improve breeding and production stocks to increase efficiency and decrease losses, with the goal of producing more food with fewer resources. While broader application of classical selective breeding can make valuable contributions to genetic improvement, researchers and developers are applying rDNA techniques and genome editing (GnEd; see Glossary of Terms) to achieve outcomes that would not otherwise be possible, as well as combining GnEd techniques with genomic selection (Meuwissen 2007) to achieve genetic advances much more quickly than could be achieved by conventional breeding methods.

### Genetic modification using rDNA techniques

Historically, the term ‘genetically modified’ (GM) has referred to organisms, including animals, that are produced by classical gene transfer methodologies in which an introduced recombinant DNA (rDNA) construct integrates randomly into the host genome, or by breeding of transgenic founders that transmit that rDNA construct to their descendants. It has been over forty years since the first report of using rDNA technology to modify animal DNA, mouse embryos expressing an SV40-thymidine kinase construct (Gordon et al. 1980). Following soon after, growth hormone transgenic mice growing to large size were produced, demonstrating that traits relevant to agriculture might be purposefully approached (Palmiter et al. 1982, 1983). This led to considerable subsequent research with larger, agricultural animals (Hammer et al. 1985, Fig. 1). Examples of such applications include cattle modified for improvement of milk composition (Liu et al. 2017) and mastitis resistance (Donovan et al. 2005); pigs for growth rate (Purcell et al. 1990), milk composition (Tong et al. 2011), and phytate utilization (Forsberg et al. 2003); sheep for growth rate (Adams et al. 2002) and wool growth (Damak et al. 1996), chickens for disease resistance (Lyall et al. 2011), and fishes for growth rate (Du et al. 1992) and disease resistance (Dunham et al. 2002). Despite the development of many promising GM animals, only one GM animal food product has reached the marketplace, the AquaBounty “AquAdvantage” salmon. The salmon are now being produced in the United States after a U.S. production facility was approved by U.S. Food and Drug Administration (2018) and a ban on import of fertilized eggs was lifted in 2019. In May 2021, Brazilian regulators approved the AquAdvantage salmon for sale and consumption in that country. Field trials have been completed in Argentina, and Chinese authorities have authorized AquaBounty to conduct regulatory trials.

**Fig. 1:**
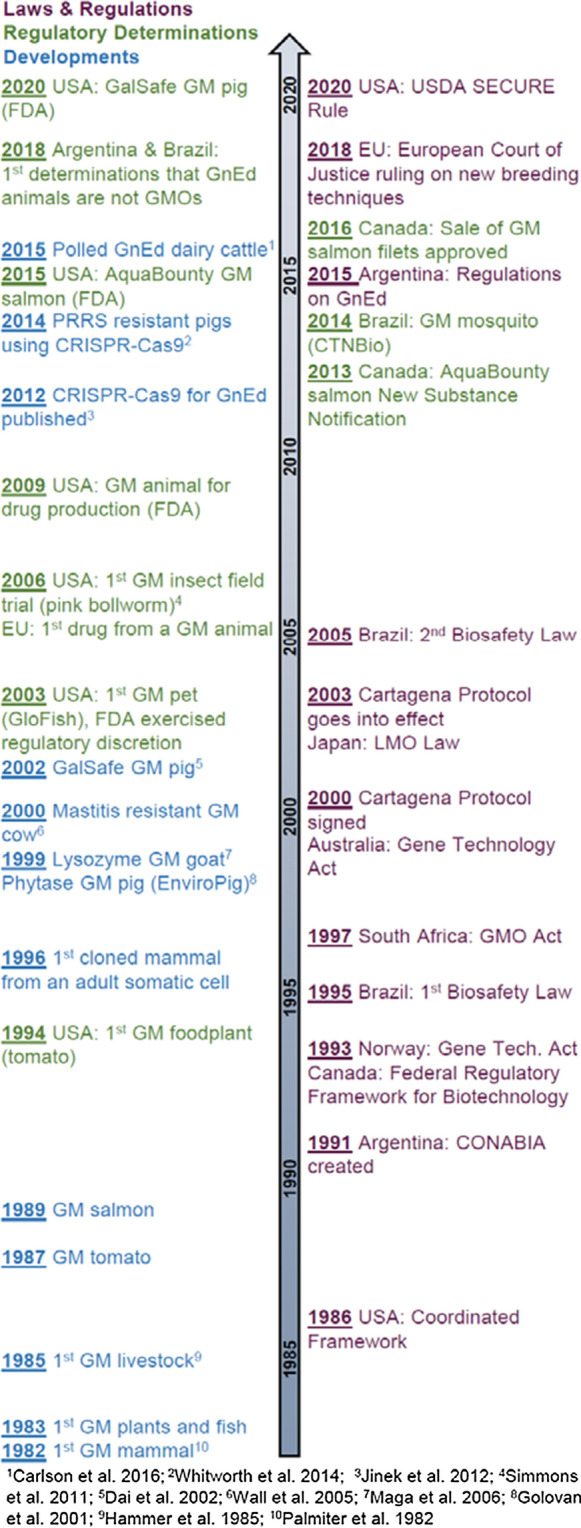
Timeline of major scientific and regulatory developments of animal biotechnology for agriculture. Note years for developments are by date of modification, if reported, or otherwise by publication year (see footnote references)

Many promising GM animals have not achieved regulatory approval. For example, the EnviroPig was a transgenic pig synthesizing phytase in the salivary glands and secreting active enzyme in the saliva, enabling them to utilize practically all the phosphorus in cereal grains and soybean meal and to excrete fecal material containing 60% less phosphorus than non-transgenic pigs (Golovan et al. 2001; Forsberg et al. 2003). It received environmental approval in Canada for commercial production (Government of Canada 2012), but the application for food approval in Canada and the United States was discontinued in 2012. The developer cited the expense, length, and uncertainty of the approval process as key factors for ending the program.

The U.S. Food and Drug Administration (2020) recently approved an rDNA construct inserted into a line of domesticated pigs using homologous recombination, referred to as GalSafe pigs. These GM pigs were developed for biomedical uses and are approved for use both as food and for use in human therapeutics. Other GM animals still in the R&D pipeline include pigs expressing bovine α-lactoglobulin in milk to improve pig growth and health (Bleck et al. 1998) and goats expressing lysozyme in milk, which may have human health benefits as well as increased shelf life (Maga et al. 2006; Cooper et al. 2013). While classical gene transfer methods were effective for the development of these animals, more so with the advent of somatic cell nuclear transfer or cloning, integration rates were relatively low. Typically, only one to several percent of microinjected embryos subsequently expressed the inserted gene, and integration of the construct was at random sites across the genome, raising concern for insertional mutagenesis and animal welfare.

### Advent of genome editing

Techniques have recently been applied to agricultural animals to alter a DNA sequence at a targeted site in the host genome using a genome editor to delete, insert, or change nucleotides in a DNA sequence. Genome editors are a set of molecular tools that allow researchers to modify the genome with a higher rate of success and higher precision than previous rDNA technologies. While different genome editors are available, such as zinc finger nuclease (ZFN) and transcription activator-like effector nucleases (TALENs), the newer “clustered regularly interspaced short palindromic repeats” associated nuclease system (CRISPR/Cas9) has become the choice of most developers. CRISPR/Cas9 utilizes small RNAs that recognize a specific DNA sequence to guide the nuclease to the correct target in the genome and to generate a DNA cleavage. The break in the DNA molecule will be repaired by non-homologous end-joining or homology-directed repair, which results in mutation via deletion, insertion, or changing of one or more nucleotides at a targeted site. With homology-directed editing, a DNA template directs the editing of the sequence for a target mutation (Doudna and Charpentier 2014). The CRISPR-Cas9 system is easy to design and assemble, can target any gene in the genome, can work with different nucleases to induce single- or double-strand breaks and can edit single or multiple sites simultaneously when using multiple guides (Chandrasegaran and Carrol 2016; Wu et al. 2018). Homologous recombination, such as the technique used to produce GalSafe pigs, occurs at a rated estimated at one in a million cells. The rate of success for editing the genome of, for example, cattle embryos can range from 13% to higher than 90% (Owen et al. 2020; Camargo et al. 2020), varying with the approach employed, the targeted gene and the type of genetic modification intended (e.g., deletion, substitution, or insertion). Reported rates of success across livestock species have ranges from 12.5 to 100% (Tan et al. 2013; Navarro-Serna et al. 2020). Moreover, CRISPR/Cas9 can be used to modulate gene expression when combined with transcription regulators (Pickar-Oliver and Gersbach 2019). All of these features make the CRISPR/Cas9 system more flexible, creating more possible applications than other GnEd tools.

Promising applications of genome-edited agricultural animals are in the research and development pipeline. While GnEd techniques have been used to create transgenic or GM animals, most developers and breeders have focused on creating cisgenic animals that could have been created via conventional breeding methods, albeit much more slowly and with less control over the breeding process. The research and developer communities seek to bring these GnEd animals forward to agricultural production and the consumer marketplace. These communities would benefit by learning from experiences with GM plants and animals and engage the regulatory community, producers, and public proactively to ease access to the marketplace, utilizing effective communication strategies to inform useful dialogue as opposed to endless debate.

### Workshops on regulatory approaches for agricultural applications of animal biotechnology

In 2011, Argentina held the First International Workshop on the Food and Environmental Safety Assessment of GM Animals in Buenos Aires. Since that time, two additional in-person international workshops have been held in Brasilia, Brazil and Charlottesville, VA, USA to discuss the range of issues associated with animal biotechnologies and their regulation (https://sites.google.com/a/vt.edu/animalbiotechresources/third-annual-2017). With the ongoing COVID-19 pandemic, a planned in-person meeting was not possible in 2020. Instead, those involved with organizing previous in-person workshops offered virtual workshops for the animal biotechnology research, breeding, and regulatory communities over seven sessions from September to December 2020 (https://sites.google.com/a/vt.edu/animalbiotechresources/2020-online-workshops). Five sessions combined presentations and discussion panels that covered topics including food safety assessment, environmental risk assessment, GnEd regulatory approaches, recent developments in animal biotechnologies and animals in the research pipeline, effective communication, and fostering of market and public acceptance. Two sessions consisted of smaller regional breakout groups with active dialog covering topics such as communication, regulatory approval, marketing and trade, and region-specific issues. The number of participants per webinar session varied from 101 to 277, with somewhat fewer participants attending the breakout groups; a total of 497 individuals from 51 countries took part across the seven sessions. The session on regulatory approaches for GnEd animals attracted the most participants, demonstrating the global interest in this topic. In this review, we summarize the topics presented and discussed, with additional context to inform the wider community of biotechnology researchers, developers, breeders, and regulators.

## Delivering the promise of genome editing

### Status of genome editing in food animals

The rise of genome editors opened new opportunities for genetic improvement of livestock, especially when combined with information gathered through sequencing of livestock genomes. In a given animal breed, GnEd technologies can: (1) promote the introgression of a gene or allele associated with a favourable trait found in other breeds, without requiring crossbreeding and unintended introgression of linked traits (Tait-Burkard et al. 2018); (2) modify quantitative traits associated with several quantitative trait nucleotides to increase the frequency of favourable alleles (Jenko et al. 2015); and (3) delete genes with deleterious or undesirable effects (Whitworth and Prather, 2017). Thus, genome editors can be applied to improve meat and milk composition, improve animal welfare and climate resilience, increase disease resistance, and enhance agricultural productivity.

Genome editors can be used to improve the welfare of livestock. For cattle, horns present risks to farmers and other animals, and farmers frequently remove horn buds from young calves using chemicals or a hot iron. The introgression of a *polled* allele from a naturally hornless polled breed into the genome of embryos from a horned breed resulted in hornless, or polled, calves (Carlson et al. 2016), with no need for subsequent disbudding. For pigs, several genes are being targeted to reduce or eliminate boar taint in pork (Telugu 2020), which could eliminate the need to castrate male pigs.

Genome-edited traits could help cattle to be more acclimated to warmer temperatures, improving animal welfare and livestock productivity in the tropics. A black coat absorbs more solar radiation and retains more heat than a white coat, contributes to increased body temperature (Hilmann et al. 2001), and can impair production and reproductive functions (King et al. 1988). Dilution of black color in Holstein cattle was achieved by the introgression of mutations in the pre-melossomal protein 17 gene, resulting in a calf with a grey and white coat (Laible et al. 2020). Some cattle breeds present a slick, short-hair phenotype due to mutations in the prolactin receptor (*PRLR*) gene, which improves the animal’s ability to manage body temperature (Porto-Neto et al. 2018). Introgression of such *PRLR* alleles can generate animals with slick, short hair (*SLICK*) and increase heat tolerance in thermally sensitive breeds (Hansen 2020), such as Angus and Holstein.

Resistance to livestock diseases also can also be enhanced by GnEd. Porcine reproductive and respiratory syndrome (PRRS) is a viral disease that affects the respiratory and reproductive systems of swine and results in large economic losses for the pork production industry worldwide (Neumann et al. 2005). Deletion of the *CD163* gene, which encodes a cell-surface protein that mediates the entry of the PRRS virus into the host cells, or the deletion of its scavenger receptor cysteine-rich domain 5, conferred resistance to the infection (Whitworth and prather 2017; Burkard et al. 2018; Xu et al. 2020). The PRP gene has been knocked out to provide resistance to all sources of prion disease, including "mad cow” disease (Richt et al. 2007). Park et al. (2020) used CRISPR/Cas9 to introduce novel *PRNP* (prion gene) allelic variants into cattle embryos that have been shown to provide resilience to human prions. Genome editing can be applied to enhance resilience to important diseases of poultry, such as avian leukosis (Lee et al. 2017).

Genome editing may be utilized for genetic alterations affecting production, product quality or creating foods with different nutrient profiles. Cows lacking expression of the beta-lactoglobulin protein, one of the main allergens in cow milk, were generated by deleting or silencing the gene that encodes the protein (Wei et al. 2018). Sheep with deletion of the myostatin gene exhibit heavier weight than wild-type individuals (Crispo et al. 2015).

With high fecundity and external fertilization and development, fishes offer an attractive system for GnEd. CRISPR/Cas9 editing is being applied to at least 11 aquaculture species to achieve reproductive confinement, increase growth rate, improve disease and pest resistance, increase omega-3 fatty acid content, or affect pigmentation (Zhu and Ge 2018; Gratacap et al. 2019). For example, GnEd has been applied to knock out the myostatin gene of common carp (Zhong et al. 2016), red sea bream (Kishimoto et al. 2018), olive flounder (Kim et al. 2019), and yellow catfish (Zhang et al. 2020) to achieve greater muscling and filet size. To achieve reproductive confinement of cultured Atlantic salmon, Wargelius et al. (2016) deleted the *dnd* gene to induce germ-cell ablation, with concurrent deletion of *slc45a2*, yielding albinos as a phenotypic marker.

There are other opportunities to apply GnEd to improve quantitative traits that are controlled by single genes, and many other opportunities may emerge with the identification of new targets and their effects. Progress in the science and practice of GnEd may allow introduction of multiple edits with positive effects on quantitative traits controlled by many genes, which in turn may result in a greater response to selection when combined with genomic selection in livestock breeding programs (Jenko et al. 2015; Georges et al. 2019).

### Moving towards practical application

Work on GnEd animals has moved beyond proof of principle toward practical applications that would optimize livestock for microenvironments and purposes faced by millions of small farmers. Commercialization of GnEd animals could improve animal well-being, provide economic benefits to farmers, and promote sustainability. Ideally these breeding technologies will be developed with the engagement of stakeholders, including farmers and consumers (Brody 2020). Bringing forward the achievements of research and development (R&D) to commercial application will require partnerships among universities, government research institutes and the private breeding and production sectors. For example, application of GnEd to introduce indels into the bovine β-lactoglobulin gene is going forward through collaboration between INTA (Instituto Nacional de Tecnología Agropecuaria) and the Universidad de San Martin in Argentina. Acceligen is actively working on 12 traits in cattle, swine, and fish (Sonstegard 2020). Noting that two-thirds of global cattle population is held by 300 million small holders, Acceligen and collaborators aim to generate dairy animals that will bring about significant and sustainable production gains through immediate access to improved heat-tolerant and disease-resistant dairy cattle, particularly for African dairy production systems. Acceligen (USA), Kheiron Biotech (Argentina), and TransOva Genetics (USA), with support from the Bill and Melinda Gates Foundation, are collaborating to combine valued traits from the Brazilian Gir (a breed tolerant of tropical conditions with origins in India) and Holstein (a breed with high milk production) cattle. Targeted genes and traits will include *polled*, *SLICK*, and trypanosome parasite resistance, with the aim of dramatically increasing production in South American (Sammartino 2020) and African herds. The Mzima cow project of the Centre for Tropical Livestock Genetics and Health, with support from UKAid and the Bill and Melinda Gates Foundation, is aimed at producing GnEd cattle expressing a synthetic trypanolytic factor derived from the baboon *ApoLI* gene to make them resistant to trypanosomes, the parasite responsible for African sleeping sickness (Kemp 2020). Genome editing of polo horses has been undertaken by the Proinvesa Group of Argentina (Knapton 2017; Sammartino 2020). The swine production industry is interested in applying GnEd to address the multi-billion dollar impacts of the PRRS virus (Whitworth et al. 2014, Burkhard et al. 2018, Xu et al. 2020, Brody 2020) and to improve animal welfare by eliminating the need for castration to prevent boar taint in pork (Telugu 2020). In poultry, marker-assisted sex selection has been proposed as a replacement for culling of day-old male chicks in the egg layer industry. Inserting the marker gene into the male-determining sex chromosome would enable male eggs to be identified and removed at point of lay. The female eggs, not carrying the marker gene, would be incubated and hatched as hens to lay eggs for food production and hopefully would not be regulated as GM in Australia, as the hens would be “null segregants” (Doran et al. 2016a), though such determinations might vary among jurisdictions. Other practical applications in poultry include introduction of disease resistance to improve animal welfare and reduce potential for zoonotic spread of diseases (in particular, avian influenza), and improvement of food safety by deletion of allergen-encoding sequences (Doran et al. 2016a, b; Oishi et al. 2016).

## Food safety aspects of genetically modified and genome edited animals

Technical methods applied in animal biotechnology have evolved, and GnEd techniques are becoming widely applied to develop animals with new traits. Whatever the biotechnological approach, there is a widely perceived need to assure the public of the food safety of products from genetically modified animals. National and supranational bodies have worked to develop policies for that purpose (see Fig. 1 for timeline); many have done so for organisms developed using classical rDNA technology methods. Additionally, most countries are signatories of the Convention of Biological Diversity (CBD) and the Cartagena Protocol on Biosafety (CPB), a regulatory framework generated to govern transboundary movement of living modified organisms (LMOs) and to ensure human health and environmental safety. The development of GnEd techniques is relatively recent, and therefore, many countries and supranational bodies have yet to determine their regulatory posture. As noted below, the majority of countries with regulatory policies for GnEd organisms distinguish between GM and GnEd products based on the definition of LMO established in the Cartagena Protocol on Biosafety (Secretariat of the Convention on Biological Diversity 2000). In this section, we review issues pertinent to food safety assessment and associated policies for oversight of GM and GnEd animals as reported in the virtual workshops.

### Food safety assessment of foods derived from recombinant DNA-bearing animals

The safety of foods derived from GM and GnEd animals is important to consumers and likely will prove a critical issue for adoption of such foods. How should regulatory authorities approach food safety assessment? The CODEX Guidelines for the Conduct of Food Safety Assessment of Food Derived from Recombinant-DNA Animals (FAO 2008) is a highly influential document informing the development of national food safety assessment policies. The scope of the Guidelines is restricted to food safety and nutritional issues and to foods from animals with a safe history of use as sources of food. They are designed for food derived from recombinant-DNA (rDNA) bearing animals, although the approach could be applied to foods from animals altered by other techniques, including GnEd. The Guidelines embody a flexible, case-by-case assessment; comparisons of food safety are made to foods from conventionally bred counterparts, with a focus on intended and any unintended changes.

A number of changes were made to adapt the existing Codex guidelines for foods derived from rDNA plants to those from rDNA animals. Among terminology and definitions changed, the “host” is now the “recipient animal prior to the genetic modification”. Distinction is now made between the “initial rDNA animal” (i.e., the founder animal) and the “final rDNA animal used for food”; that is, some founder animals prove to be mosaics, and additional breeding is required to ensure that the insertion is germ-line transmissible, and the focus of the food safety assessment is on the final rDNA animal. The definition of “conventional counterpart” has been revised to better reflect animal breeding practices. The assessment of potential allergenicity (including the Annex) remains the same as in the Plant Guidelines. The assessment of potential toxicity has been expanded to include bioactivity, recognizing that some modifications to animals may involve the expression of bioactive substances (e.g., hormones such as growth hormone); the food safety assessment should therefore include consideration of whether such substances may be active in humans, taking into account impacts of processing and cooking. The assessment also may be informed by the health status of the rDNA animal. The general approach to the assessment of potential toxicity remains the same as the Plant Guideline. Aspects relating to compositional analysis have been changed to include reference to the choice of comparator—ideally matched in husbandry/housing conditions, age, sex, parity, lactation, laying cycle, and other relevant factors. Application of the Codex rDNA Animal Guideline to the products of genome editing is yet before us. While only limited examples of rDNA animals for food use exist, applying the Codex Animal Guideline is essentially no different than applying the well-accepted Plant Guideline.

### Regulatory approaches for food safety of genome edited animals

Unlike GM methods that rely on random insertions of rDNA (often from other species), GnEd enables precise changes equivalent to those that could be obtained by conventional breeding techniques. The fact that GnEd uses biotechnology but allows generation of conventional products has raised challenges from the regulatory point of view. Hence, government policies have been generated, reviewed, or adapted to guarantee biological safety while enabling innovation (ECLAC et al. 2019).

Underlying any regulatory policy are the definitions of regulated articles, which thereby define the scope of regulation. Technical definitions underlying existing biotechnology regulatory policies may need to be updated to encompass new breeding technologies, such as GnEd. For example, Food Standards Australia New Zealand (FSANZ), a binational government agency, adopted standards for assessing safety of foods produced using classical gene transfer methods in 1999. Noting that it was unclear whether foods derived using newer GnEd methods are captured using these definitions, FSANZ (2019) recently completed a review of the safety of foods derived from new breeding technologies (NBTs). Among the key findings, definitions in the existing code for ‘food produced using gene technology’ and ‘gene technology’ are no longer appropriate, as they lack clarity, are outdated, and do not reflect the diversity of techniques now in use. FSANZ found that there may be a case, based on low risk, for some foods derived from new GnEd techniques to be excluded from the requirement for pre-market safety assessment. However, there are divergent views among stakeholders about the acceptability and risk of foods derived from new biotechnologies and how best to regulate them. These findings led to recommendations for updating definitions to better accommodate new and emerging technologies, increasing clarity regarding what foods are captured for pre-market approval, and regulating foods derived from new technologies in a manner commensurate with the risks posed. Public consultations were expected to begin in 2021.

Different approaches to definition of new animal breeding technologies and oversight of food safety of associated products have been adopted among countries and groups of countries. Most countries (the exceptions being the United States and Canada) have GMO laws, and the definition of GMO or LMO determine whether a particular product, technology or type of modification is under the jurisdiction of that country’s GMO regulations. The respective countries and international unions differ in terms of legal authorities (often Codex Alimentarius—FAO and WHO 2021) and lead agencies (often a food safety agency or an agency within the ministry of agriculture). Comparisons and contrasts are summarized in Table 1. Among countries that have taken any action, Argentina was first and in 2015 approved Resolution 173/15, which established a procedure to determine whether a product obtained using NBTs would be subject (or not) to the existing GM regulation. If a GnEd animal did not contain foreign DNA (i.e., was not transgenic), then it would not be considered a GMO and would be regulated as a conventionally bred animal. In 2020, the Biosafety Commission worked on updating the regulations for products obtained through NBT. Finally, the updated resolution (Resolution 21/2021, see: https://www.boletinoficial.gob.ar/detalleAviso/primera/240529/20210208?busqueda=1) was made official in 2021, covering plants, animals, and microorganisms. In Brazil, an expert group was convened in 2015 to elaborate rules for consultation involving NBTs, and in 2018 Normative Resolution No. 16 (RN16) was approved and adopted by CTNBio, which referred to Precision Breeding Innovative Techniques (TIMP). The RN16 considers that TIMP encompass a set of new methodologies and approaches that differ from the transgenic genetic engineering strategy, as it results in the absence of recombinant DNA/RNA in the final product. Examples of TIMP are presented in Annex 1 of RN16 and included (but are not limited to) RNA-dependent DNA methylation, site-directed mutagenesis, oligonucleotide-directed mutagenesis, RNAi for topical/systemic use, and viral vectors, with the inoculation of living organisms with recombinant viruses (DNA or RNA) expressing the genetic modification and amplification of the gene of interest through viral replication mechanisms, without modifying the host genome. The first consultations were held in the second semester of 2018 regarding genetically engineered yeasts and the genome-edited polled cattle (Nascimento 2020). A rapidly growing Nile tilapia was determined to be conventional. The application for GnEd polled cattle was withdrawn, however, after vector DNA was found in its genome. Genome-edited cattle with the polled and *SLICK* traits that do not contain the foreign vector DNA have since been determined to be conventional in Argentina.

**Table 1:** Approaches to oversight of food safety of products of animal biotechnology in selected countries and supranational groups

Country/Union	Regulatory agency	Regulatory policy for GM animals?	Consistent with Codex Alimentarius?	GM animal product approved for food?	Regulatory policy for GnEd animals?	Supporting presentation or reference
Argentina	SENASA^a^	Yes	Yes	No	Yes^b^	Maggi (2020) and Whelan (2020)
Australia/New Zealand	Food Standards Australia New Zealand	Yes, Food Standards Australia New Zealand Act 1991	Yes	No	No; Code under review	Kelly (2020)
Brazil	CTNBio^c^	Yes, Biosafety Law 11,105, 2005; also see 28 Normative Resolutions	Yes	Yes	Yes	Finardi (2020)
Canada	Canadian Food Inspection Agency, Health Canada, othersd	Yes	Yes	Yes	NA^e^	Cianciarelli (2020)
Japan	Ministry of Health, Labor, and Welfare	Yes	Yes	No	Yes	Tsuda and Ohsawa (2020)
Philippines	Department of Agriculture, Bureau of Animal Industry	No	Yes	No	No	Mingala (2020)
South Africa	Department of Science and Innovation	Yes	Yes	No	No	Groenewald (2020)
United States	Department of Health and Human Services, Food and Drug Administration; others^f,g^	Yes	Yes	Yes	Yes	Kanelakis (2020)
African Union^h^	–	NA	NA	NA	NA	Nengomasha (2020)
European Union	European Food Safety Authority	Yes	Yes	No	No	EFSA (2012)

^a^Servicio Nacional de Sanidad y Calidad Agroalimentaria

^b^Updated policy under consideration

^c^National Biosafety Technical Commission

^d^Others as appropriate to application, may include Environment and Climate Change Canada, Department of Fisheries and Oceans, Agriculture and Agri-Food Canada, Global Affairs Canada, and Innovation, Science and Economic Development Canada

^e^Canada’s regulations are product-based. The method of genetic modification does not determine whether a safety assessment is required; 'novelty' of product is regulatory trigger for pre-market assessment. Canada has a Proposed new guidance for Novel Food Regulations focused on plant breeding

^f^U.S. Department of Agriculture (USDA), Animal and Plant Health Inspection and Food Safety and Inspection Service released an Advanced Notice of Proposed Rulemaking in December 2020 that may result in a shift of regulatory jurisdiction for some products

^g^USDA, Food Safety and Inspection Service is responsible for the final safety determination and labeling for certain products, such as meat

^h^A union of 55 African member states aimed at promoting economic and political integration. Strategy and advisory role, rather than regulatory

The Japanese government in 2019 defined GnEd products derived by modifications of the SDN-1 type (i.e., directed mutation without using a DNA sequence template) as not representing LMOs according to the Japanese Cartagena Act (Tsuda et al. 2019; Tsuda and Ohsawa 2020). In such cases, the government requests that developers provide information on the development processes for the purpose of accumulating knowledge related to GnEd of organisms and agree to publish part of that information on the Japan Biosafety Clearing House website. Within the European Union, a GM organism is defined as having genetic material that has been altered in a way that does not occur naturally by mating or natural recombination. Taking an approach different from that of most other countries, the Norwegian Biotechnology Advisory Board has proposed a three-tiered system, with the tier depending on the risk category and type of genetic alteration (Brattlie et al. 2019). Organisms with “foreign” DNA or transgenic alterations would require standard GMO assessment. Those that could arise via conventional breeding would require only notification (with confirmation). A third category of expedited assessment would exist for organisms with other species-specific genetic changes. The proposal is currently under discussion (Holst Jensen 2020). The interpretation in several EU countries before July 2018 was that organisms with CRISPR/Cas9-induced point mutations are *not* GMOs; however, the European Court of Justice in July 2018 ruled that GnEd organisms *are* GMOs since the genomic DNA has been altered. Notably, chemical- and radiation-induced mutagenesis of crop plants was recognized under this definition but excluded on the grounds of a “history of safe use” of such technology, an exclusion not afforded the more precise technology of GnEd. South Africa is considering a governance framework for GnEd proposed by the Academy of Science of South Africa (2017) that advocates a product-based trigger where products of NBTs are classified either as GMOs that will be regulated under GMO Act 15 of 1997 or as non-GMOs that will not be subject to regulation under the Act and would be regulated as conventional products (Groenewald 2020). Clearly, not all countries have express policies for GM animal products, and only a few have taken up issues posed by GnEd animal products. Indeed, because most countries have not received applications for food products derived from GM or GnEd animals and have not readied the relevant policy frameworks, regulatory issues may delay regulatory decisions when such applications ultimately are submitted.

While many countries or supranational authorities have existing systems for ensuring the safety of foods derived from animals (Abley 2020), regardless of the genetic background, the food animal production industry emphasizes that it routinely conducts additional testing and monitoring to assess both food safety and process control (Alvarado 2020). Considerations unique to GM or GnEd products might include assessment of allergenicity of any unique gene products and compositional analysis.

### Regulatory process and commercialization of genome-edited animals

Regulatory policy, particularly the definition of what is a regulated article, have important bearing on the progress of a product through the regulatory pathway and hence on the prospect that GnEd animals will come to production in agriculture. We illustrate this influence with case studies of regulatory experiences in selected countries.

In Argentina, seven introduced traits in GnEd animals, including four traits for cattle, one equine, one swine, and one fish, have been presented to regulatory authorities. While the cattle are at the R&D stage, a GnEd fish developed by AquaBounty is potentially ready for production. The Nile tilapia FLT-01 line exhibits increased fillet yield by deletion of the endogenous myostatin gene through a 26-bp deletion creating an early stop codon. Loss of function of that negative regulator of muscle growth leads to increased muscle mass, greater weight, and greater fillet yield than its unedited counterpart. The GnEd fish was created using microinjection with nuclease mRNA; no introduction of DNA was involved. The final product is homozygous for the 26-bp deletion. There are no off-target sites of modification. Critical in regulatory consideration of the FLT-01 fish was lack of new genetic material or unwanted integration of plasmid DNA in the final product. In particular, it does not contain a new combination of genetic material in the genome generated by the application of modern biotechnology, and hence is not covered by the definition of a regulated article under Res. 763 under the Cartagena Protocol. Hence, under Argentine Resolution 173/15—New Breeding Techniques, this fish is not a GMO. Brazil made a similar determination for this GnEd fish in August 2019.

A major global animal GnEd milestone was reach in 2021 when the first food from GnEd animals was marketed in Japan. In 2021, the Ministry of Health, Labor and Welfare (MHLW) determined that two GnEd fishes with increased edible muscle (GnEd myostatin knockouts in red sea bream *Pagrus major* and tiger puffer *Takifugu rubripe*) are not GM and therefore are not subject to a requisite GM food safety review (MHLW 2021). A Kyoto-based start-up, Regional Fish Institute, Ltd., submitted the notifications to MHLW and the Ministry of Agriculture, Forestry and Fisheries to market these fish, which were the result of a collaboration between Kyoto and Kinki Universities (Asahi Shimbun 2021b, a, Yomiuri Shimbun 2021b, a).

The experience of a GnEd polled cattle trait in the Brazilian regulatory process provides a cautionary tale regarding product development and the regulatory process. Under Brazilian Biosafety Law 11.105, a GMO is an organism whose genetic material has been modified by any genetic engineering technique. Genetic engineering is defined by Brazil as the activity of manipulating DNA or RNA recombinant molecules, and recombinant DNA or RNA molecules are molecules manipulated outside live cells through changes made to natural or synthetic DNA or RNA segments that can multiply in a live cell. A petition was submitted to CTNBio for commercialization of semen of a polled bull produced using GnEd techniques by Recombinetics (USA) and Agropartners (Brazil). CTNBio analyzed and approved the GnEd cattle as non-GMO in October 2018. However, after the U.S. Food and Drug Administration analyzed the genome sequence of the bull in 2019 and unexpectedly found transgenic plasmid DNA sequences, the animal was then considered to be a GMO under Brazilian regulations. While the presence of the specific transgene or plasmid sequence in the genome of this GnEd animal was unlikely to pose a food safety concern, it would make the animal transgenic, and Recombinetics and Agropartners withdrew the application for further consideration by Brazilian regulators.

**Fig. 2:**
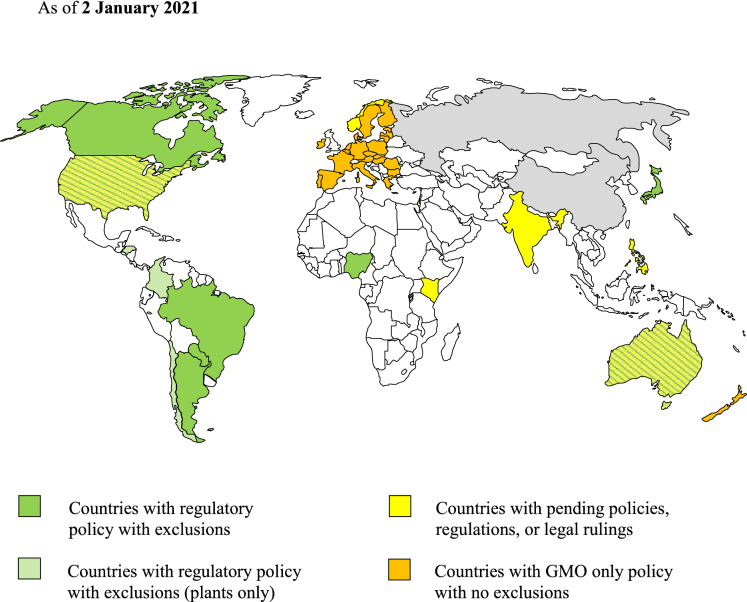
Overview of national or supranational regulatory regimes for GM or GnEd animals

From the developers’ viewpoint, GnEd offers flexible, fast, relatively inexpensive tools, which are potentially accessible to a broader range of developers. However, the success of the technology for agricultural animal applications will depend on the expense of passage through regulatory systems (Nesbitt 2020). To foster innovation, regulatory systems must: (1) ensure animal health and welfare, food safety, and environmental health, (2) be science-based and risk-proportionate, (3) have clear, transparent processes and evaluation criteria with predictable timelines, (4) allow animals to be treated as conventional farm animals, and (5) be globally aligned. If regulatory systems do not meet these criteria, the consequences may include movement of R&D activity and commercialization to countries with more amenable regulatory regimes (Fig. 2). For example, Van Eenennaam et al. (2019) termed the proposed U.S. animal drug regulatory approach for GnEd food animals as “not fit for purpose”, and Ledford (2019) cited examples of American developers of GnEd animals moving their work elsewhere. Hess (2019) described Argentina, Brazil and Canada as outpacing United States in development of genome-edited animals.

## Environmental safety aspects of regulations

### Environmental risk assessment

**Fig. 3:**
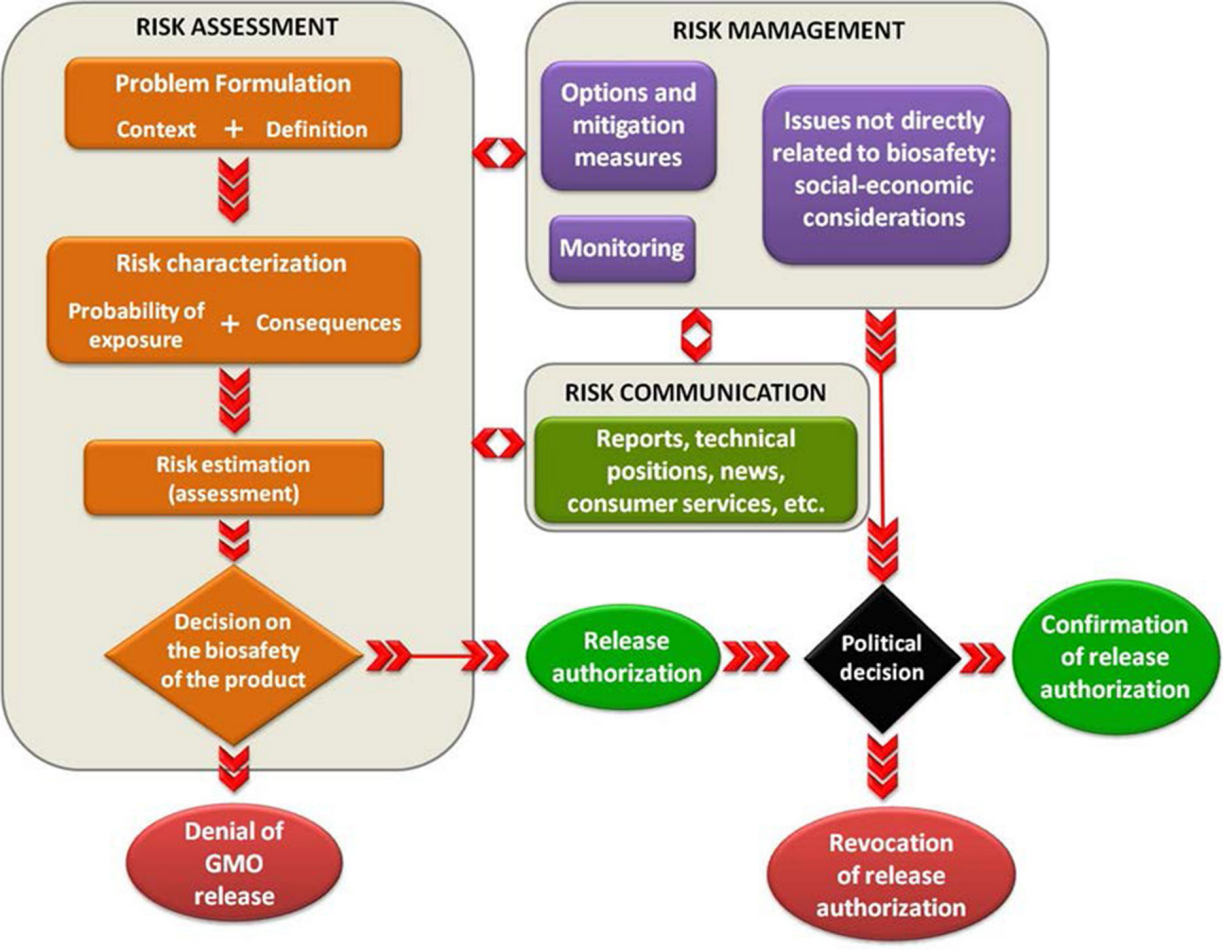
An overview of the environmental risk analysis process (Roca et al. 2015; Andrade 2020)

A GM or GnEd animal will become incorporated into routine agricultural production only with demonstration that production can go forward in a safe manner in a particular environmental context. Environmental risk can be assessed and managed by applying the formal risk analysis process (Fig. 3, Andrade 2020), a general approach historically developed for epidemiological or chemical exposures that has been extended for application to GM plants and animals (Hilbeck and Andow 2004; Hilbeck et al. 2006; Kapuscinski et al. 2007; Roca et al. 2015). In the risk assessment context (NRC 1996, 2002), a harm is defined as an ecological perturbation resulting in negative impacts to a receiving ecosystem. A hazard is defined as an agent—the GM or GnEd animal—that has the potential to produce harm. Risk is defined as the likelihood of harm resulting from exposure to the hazard. Risk, *R*, is estimated as the product of the probability of exposure, *P*(*E*), and the conditional probability of harm becoming realized given that exposure has occurred, *P*(*H*|*E*). That is, *R* = *P*(E) × P(*H*|*E*). The steps in risk analysis, then, are to: (1) identify the protection objectives; (2) identify hazards that might lead to harms in the context of protection objectives; (3) define what exposure means for a GM or GnEd animal and assess the likelihood of exposure, *P*(*E*); (4) quantify the likelihood of harm given that exposure has occurred, *P*(*H*|*E*); and (5) multiply the resulting probabilities to yield a quantitative estimate of risk. Exact probabilities of risk are difficult or impossible to determine for all types of possible harms. Indeed, it is unlikely that all possible harms would be known a priori, particularly for any indirect causal pathways, and quantitative estimation of *P*(*H*|*E*) is difficult. Hence, it may be necessary to classify levels of concern regarding likely genetic impacts posed by production of GM or GnEd livestock into qualitative categories ranging from “low” to “high” (Table 2). For each identified hazard, the qualitative class of risk from “negligible” to “high” is determined by the likelihood of exposure or harm (“very low” to “very high”) and the magnitude of the consequence (“marginal” to “major”). Usually only the negligible risks are considered acceptable.

**Table 2:**
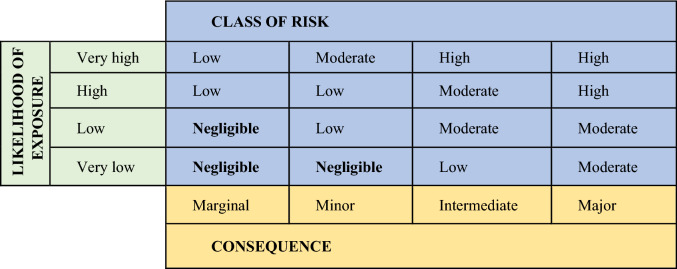
A qualitative approach to environmental risk assessment (Andrade 2020)

The class of risk for each hazard, from negligible to high, is determined from the likelihood of exposure and magnitude of the consequence as defined by science

The yellow in the original version made clear that Consequence related to the Marginal, Minor, Intermediate and Major descriptors in the row above it

Risk assessment (Fig. 3) involves both scientific and values-related considerations conducted within the context of interactions among a range of stakeholders (Andrade 2020). This involvement will bring all existing knowledge into the process, make the process transparent to stakeholders, and enhance the understanding and acceptance of the outcome of risk analysis. Stage I involves identifying the problems at hand, engaging stakeholders, identifying potential harms, risk pathways and assessment methods (Table 3). Stage II is the risk assessment itself, leading to estimating the likelihood that harm will become realized should a proposed action be taken. Upon estimation of that risk, a decision is faced as to whether the risk is acceptable. If it is acceptable, the decision may be made to go forward. If the level of risk is unacceptably high, risk management measures would be identified and residual risk quantified, and the decision of whether to go forward would again be considered.

**Table 3:** Framing of problem formulation for environmental risk assessment for three types of GnEd or GM animals in Brazil (adapted from Andrade 2020)

Animal	Biodiversity protection goal	Biology of organism	Receiving environment	Gene construct	History of safe use
GnEd polled cattle	No obvious protection goal	Non-native No sexually compatible species Dispersion under control Moderately invasive Not relevant for wildlife food chain	Agricultural systems	CRISPR/CAS9 leading to gene silencing	To some extent, yes
GM fast-growing tilapia	Native river species	Non-native No sexually compatible species Dispersion under poor control Very invasive Relevant for wildlife food chain	Ponds, rivers and lakes	Transgene constitutively expressing a growth hormone	None
GnEd snail for gene drive-mediated population suppression of an invasive population	No obvious protection objectives, unless it does not function as expected: if so, then native river species	Non-native No sexually compatible species Uncontrolled dispersal, very invasive Not relevant for wildlife food chain	Agricultural areas	Gene-drive construct for male-only phenotype/ fluorescence	None for snails

### Regulatory approaches in different countries

For oversight of environmental safety aspects of biotechnology, the most influential regulatory instrument is the Cartagena Protocol on Biosafety, an international agreement concluded and adopted under the framework of the Convention on Biological Diversity (CBD). The CBD is aimed at “ensuring safe transfer, handling and use of LMOs resulting from modern biotechnology that may have adverse effects on the conservation and sustainable use of biological diversity, also taking into account risks to human health, and focusing on transboundary movements” (Secretariat of the Convention on Biological Diversity 2000).

Under the Cartagena Protocol, an LMO (which is roughly equivalent to GMO in more common usage) means any living organism that possesses a novel combination of genetic material obtained through the use of modern biotechnology. "Modern biotechnology" is then defined as the application of: *a*. in vitro nucleic acid techniques, including recombinant deoxyribonucleic acid (DNA) and direct injection of nucleic acid into cells or organelles, or *b*. Fusion of cells beyond the taxonomic family, that overcome natural physiological reproductive or recombination barriers and that are not techniques used in traditional breeding and selection. These definitions were developed during the time when classical GM organisms were at issue; their relevance to GnEd animals is unclear. The applicability of the framework to different classes of modified organisms is still under discussion, particularly regarding clarification of the scope of regulatory definitions and international harmonization of regulations. In the meantime, some countries have adopted regulatory approaches similar to that embodied in the Cartagena Protocol (Argentina, Brazil, Chile, Colombia, Honduras, and Paraguay). Some countries offer official scientific advice similar to the Cartagena Protocol (Kenya, Nigeria, and South Africa). Some countries have regulatory pathways that yield similar end results for most SDN1 GnEd animal products (Australia, Canada, and Japan). Some countries are bound by court decisions contrary to official scientific advice and national regulatory decisions (European Union and New Zealand). Not all countries are signatories of Cartagena Protocol on Biosafety, notably Argentina, Australia, Chile, Canada, and United States. Table 4 presents an overview of how individual countries and the European Union have instituted regulatory approaches incorporating environmental risk assessment of GM and GnEd animals. With its departure from the European Union, the United Kingdom is considering new policies to make it easier to test and commercialize GnEd crops and livestock (Stokstad 2021). Additionally, the African Union InterAfrican Bureau for Animal Resources (AU-IBAR) is promoting regulatory cooperation and development of harmonized guidelines among its member states (Nengomasha 2020).

**Table 4:** Comparisons and contrasts of regulatory approaches related to environmental risks of animal biotechnology among countries with GM or GnEd regulations in place

Country/union	CPB^a^ party?	GM animals authorized?	GnEd regulatory approach in place?	GnEd animals authorized or deemed conventional?	Responsible Agency for animal biotech authorization	Supporting presentation
Argentina	No	No	Yes	Yes	CONABIA^b^	Boari (2020); Whelan (2020)
Australia	No	No	Yes; Third Review of National Gene Technology Scheme is in process^c^	No	Office of the Gene Technology Regulator^d^	–
Brazil	Yes	Mosquito, Salmon^e^	Yes	Yes	CTNBio^f^	Garcia (2020a, b)
Canada	No	Pig, Salmon^g^	NA^h^	No	Environment Canada	–
India	Yes	No	No	No	Ministry of Environment and Forests	Majumdar and Jain (2020)
Japan	Yes	Silkworm	Yes	No	Ministry of Environment	Ohsawa and Tsuda (2020)
New Zealand	Yes	Yes^i^	Yes	Yes^j^	Environmental Protection Authority	Strabala (2020)
Nigeria	Yes	No	No	No	National Biosafety Management Agency	Omeje and Gidado (2020)
Norway	Yes	No	No	No	Ministry of Climate and Environment	Holst-Jenson (2020)
Kenya	Yes	No	Yes, awaiting publication	No	National Biosafety Authority	Ogoyi (2020)
Philippines	Yes	No	No	No	NCBP, DOST, and DENR^k^	Salces (2020)
South Africa	Yes	No	No	No	Ministry of Agriculture, Forestry and Fisheries	Rhodes and Groenewald (2020)
United States	No	Salmon, Pig, Insects^l^	Yes	No^m^	HHS/FDA, USDA/APHIS, EPA^n^	–
European Union	Yes	No	No	No	European Food Safety Authority	Schoonjans et al. (2020)

^a^Cartagena Protocol on Biosafety, a follow-on to the Convention on Biodiversity

^b^CONABIA—National Advisory Commission on Agricultural Biotechnology, within the Ministry of Agriculture, Livestock and Fisheries

^c^https://www1.health.gov.au/internet/main/publishing.nsf/Content/National-Gene-Technology-Scheme

^d^Gene Technology Act 2000

^e^Oxitec reproductively confined mosquito, AquAdvantage Atlantic salmon

^f^CTNBio, the National Biosafety Technical Commission

^g^AquAdvantage salmon

^h^Canada’s regulations are product-based. The method of genetic modification does not determine whether a safety assessment is required; 'novelty' of product is regulatory trigger for pre-market assessment under the New Substances Notification Regulations (organisms) of the Environmental Protection Act (1999)

^i^Field trials of GM animals have been approved: high casein-expressing cattle, β-lactoglobulin knockdown cattle, monoclonal antibody-producing cattle and goats

^j^Contained production of pigs for improved human immunocompatibility

^k^National Committee on Biosafety of the Philippines, Department of Science and Technology, and Department of Environment and Natural Resources

^l^AquAdvantage salmon, Gal-safe pig limited to a single biomedical facility with restrictions on rearing conditions and slaughter facility, insects with different traits for limited field trials

^m^Rulemaking in progress (U.S. Department of Agriculture—Animal and Plant Health Inspection Service 2020)

^n^Department of Health and Human Services—Food and Drug Administration, U.S. Department of Agriculture—Animal and Plant Health Inspection Service, Environmental Protection Agency

While particular policy approaches differ because of differing institutional structures and regulatory histories, they share key similarities of technical approach. Case studies of national environmental regulations illustrate contrasts among regulatory processes and sometimes also for treatment of GM versus GnEd animals. In Argentina (Boari 2020), the Secretariat of Food, Bioeconomy and Regional Development within the Ministry of Agriculture, Livestock and Fisheries is the national authority for activities involving GM animals. The Secretariat is advised by CONABIA (National Advisory Commission on Agricultural Biotechnology), SENASA (National Service for Agrifood Health and Quality) and the Undersecretary of Agricultural Markets. CONABIA carries out the environmental risk assessment for confined activities or commercial release. CONABIA members are scientists, professionals, and specialists from different institutions, both from the public and private sectors. The goal of the environmental risk assessment (ERA) is to protect biodiversity, human and animal health, and the agroecosystems. CONABIA bases its evaluation on principles of formal risk analysis. The ERA for GM animals is flexible and applied on a case-by-case basis; data must be of sufficient quality and quantity and based on scientific criteria, with assessment of risk based on use of a suitable conventional counterpart. CONABIA conducts environmental risk assessment, SENASA assesses food safety, and the Undersecretary of Agricultural Markets performs economic evaluation and assesses the impact on foreign trade. The three institutions submit a non-binding advisory report, recommending allowing or denying the proposed activities, after which the Secretariat grants or denies the authorization. For proposed confined activities, the facility’s biosafety conditions are assessed with the intent of maintaining a safe environment during and after the project, preventing mating outside the project, avoiding animals escaping, and keeping them from entering the food chain or commercialization. Cases presented for confined activities in Argentina since 2005 have included GM fish expressing a growth hormone transgene for human consumption and cattle and sheep with altered milk composition for biopharming or human consumption. For commercial release, additional consideration is given to protection of biodiversity, human and animal health and agroecosystems. There have not yet been requests for commercial release of GM animals in Argentina.

In Brazil (Garcia 2020b), CTNBio in 2016 recognized the need to evaluate New Breeding Technologies, framed upon the legal definitions in Article 3 of Brazilian Biosafety Law 11.105/2005, the Biosecurity Act. Normative Resolution No. 16—Técnicas Inovadoras de Melhoramento Genético recognizes new methodologies that differ from classical genetic engineering that result in the absence of recombinant DNA/RNA in the final product. The regulatory process begins with a consultation letter, which presents descriptions of the parental and product animals and description of the technique(s) employed, which are used to determine the legal framework for the product. The determination is made of whether the new product is a GMO subject to Brazilian Biosafety Law 11.105, or not, for which non-GMO regulations apply. The analysis is carried out on a case-by-case basis, with attention to off-target effects upon the host, and extra precaution for cases involving gene drive. Under Normative Resolution Nº 16, the first cases approved as non-GMOs included some yeasts, polled cattle (although the application was later withdrawn) and fast-growing Nile tilapia.

Regulation of animal biotechnology in New Zealand (Strabala 2020) goes forward under the Hazardous Substances and New Organisms Act of 1996. All organisms not present in New Zealand before 29 July 1998 are regarded as “new” organisms, including all GMOs. Under the Act, a GMO is any organism in which any of the genes or other genetic material: (a) have been modified by in vitro techniques; or (b) are inherited or otherwise derived, through any number of replications, from any genes or other genetic material which has been modified by in vitro techniques. Different release pathways are applied for importation into containment for GMOs that will not be further modified, development in containment for creating new GMOs, field trial in containment, and release with or without controls. Actions under any of these release pathways must meet Sect. 36 Minimum Standards. That is, the Environmental Protection Authority (EPA) would decline the application if the new organism is likely to cause significant displacement of native species in natural habitat, significant deterioration of natural habitats, significant adverse effects on human health and safety, significant adverse effect to New Zealand’s inherent genetic diversity, cause disease, be parasitic, or become a vector for human, animal, or plant disease. The EPA takes into account all information presented to it, including all public submissions and statements at hearings, and evaluates the benefits of the release against the risks. Current outdoor uses of GM animals in New Zealand include field trials of GM cattle overexpressing casein in milk and with knockdown of lactoglobulin to yield hypoallergenic milk. Genome editing technologies are considered as creating GMOs, per regulatory changes made in 2016. Approval has been given for the development of GnEd Auckland Island pigs in indoor containment for improved human immunocompatibility.

## Moving towards the market and public and consumer acceptance

### Public acceptance

The commercial success of GM and GnEd animals will depend upon public and consumer acceptance. Five key lessons can be taken from public attitudes to using genetically modification in animals in our “post-truth, post-trust, post-expert world” (Cormick 2020): (1) With the passage of time, attitudes regarding GM animals are becoming much closer to those for GM foods generally, with a lessening of high concern, and with younger people more accepting than older people. (2) Public attitudes fall into four segments ranging from highly supportive to highly unsupportive; many of the latter segment will likely never accept GM animal products. (3) The two middle segments defined as “Yes, but…” and “No, however…” are open to changing their minds and will determine what information should be shared to address public concerns. Public attitudes are best understood by analyzing these two groups. (4) Emotional attitudes are generally not driven by facts and logical arguments. (5) Trust is vital when information is contested; only 10–20% of the population is well informed on biotechnology, and most people go to social and mass media for information.

Important differences between expert and citizen perceptions of risk will affect the adoption of animal biotechnology (Frewer et al. 2016, Frewer 2020). Experts rely on technical risk assessments and use scientific argumentation, and generally do not take account of socio-economic impacts. Experts tend to balance risk against benefits, although it is not always clear how socio-economic or even technical benefits are assessed. In contrast, members of the public tend to use risk perceptions to make judgements about risk. Hence, effective risk communication must address their concerns as well as technical risk estimates. Emotional responses, as well as moral and ethical assessments, color public perception of animal biotechnology. The public wants to place trust in regulators. A meta-analysis of consumer attitudes to GM foods (Frewer et al. 2013) showed that plant-related, “general”, or biomedical applications were more acceptable than food animal-related applications. Risk perceptions associated with both plants and animals were greater in Europe than North America and Asia, while benefit perceptions were greater in North America and Asia than Europe. Moral concerns were higher in North America and Asia than in Europe. While the concerns expressed were very similar for both GM and GnEd, the motivation for applying breeding technologies was a key issue. Financial gain was not highly valued, while improved animal health, reduced negative environmental impacts, and human health were. This observation suggests a potential “tipping point” for acceptance of GnEd animals, for example, polled cattle.

Consumer perceptions of animal biotechnology in agriculture have largely proven negative, often not because of the technology itself, but rather because of what the technology represents. That is, expert and regulatory communications about GMOs have mostly been devoid of mention of values that align with those of consumers (Moore 2020). Shared values are 3–5 times more important to building trust than sharing facts or demonstrating technical expertise. Shared values regarding agricultural biotechnology would include: benefits to consumers and providing healthy, affordable food, not farmer benefits or profit; benefits to animals by improving animal health, not increasing productivity; and benefits to the environment, not increased efficiency. Hence, scientists, companies, and government officials should express such universal values in their messaging. For example, a message on PRRS-resistant pigs that “pigs that are resistant to one of the deadliest, incurable swine diseases will suffer less and fewer will die prematurely” is more effective than “modifying pigs to be disease-resistant increases profitability”. Regulators have an important role in the conversation regarding GnEd; 50% of consumers trust what regulatory authorities say about GnEd, and 63% trust regulatory authorities to explain the science regarding safe food production.

Greater attention should be directed towards understanding public perception of particular applications; an informative case study was conducted by Australia’s Commonwealth Scientific and Industrial Research Organisation (CSIRO) regarding public perceptions of applying biotechnology to prevent the culling of male chicks in the egg-laying sector of poultry production. CSIRO’s Synthetic Biology Future Science Platform (2020) engaged a sample of 1148 Australian residents in a survey of attitudes pertaining to the set of issues. Over 600 respondents had no awareness that male chicks were culled in the egg-laying industry, and approximately 900 viewed culling as problematic. Most respondents expressed belief that the new technology would reduce or eliminate the culling of male checks and perceived the advantage of biotechnology biology over the current practice. There was moderate to high support for the development of the technology and strong willingness to purchase eggs laid by hens involved in this process. Concerns were expressed regarding tampering with nature and unforeseen circumstances. Respondents felt that the science community should engage the public transparently, respectfully, and focusing on problem-solving as opposed to promoting biotechnology.

### Building trust for modern biotechnology

As noted above, regulatory systems with clear, transparent processes have important roles to play in garnering public trust and fostering innovation in animal biotechnology (Gallo 2020). For example, the Brazilian regulatory process embodies transparency to build public trust (Dagli and Camargo 2020). The key body is CTNBio, a multidisciplinary biotechnology oversight commission created under Brazilian law in 1995 and renewed in 2005 under Biosafety Law 11.105 to provide technical support and advice to the federal government regarding formulation, updating and implementation of the National Biosafety Policy on GMOs. CTNBio is comprised of 27 Ph.D.-level professionals, including 12 subject-area specialists from outside of government, six subject-area specialists from government ministries, and nine representatives of government ministries (http://www.ctnbio.gov.br). All agendas, minutes, and activities for monthly CTNBio meetings are made available to the public. Meetings are open for attendance by registered members of the public. The CTNBio president and members have appeared in a variety of media outlets to discuss GMOs, GnEd, and new products that are released for commercialization. CTNBio meets regularly with the scientific community to promote transparency with researchers. While the question remains whether transparency is enough to build public trust and there have been some chaotic public meetings, applications of animal biotechnology are being adopted in Brazil to a greater degree than in other countries.

### Market readiness

Adoption of animal biotechnology will depend upon the readiness of all participants in the value chain from producers to farmers to distributors, collectively the market. The readiness of the market will vary among regions.

Noting that farmed Atlantic salmon has a large international market with an inefficient supply chain, AquaBounty cites the environmental benefits of producing GM salmon in indoor recirculating systems near key markets. Their consumer research (Walton 2020) shows that 53% of respondents’ first impression of GMOs related to food are neutral to very positive, 60% are neutral to very likely to purchase products they buy regularly if labeled as GMO, 70% + are neutral to very likely to purchase products that they buy regularly if labeled with the U.S. Department of Agriculture bioengineered disclosure symbol, 81% reacted as neutral to very positive to the AquaBounty and AquAdvantage story and product attributes or benefits, and 70% are likely to purchase and try AquAdvantage salmon at least once. These results suggest that the U.S. market is ready for marketing of this particular GM product.

South America presents an interesting perspective on market readiness for animal biotechnology (Garcia 2020a, b). Classical animal assisted reproductive technology methods (e.g., artificial insemination, embryo transfer, in-vitro fertilization, cloning), are well accepted and adopted if they are economically sustainable. GM livestock was not at issue, and there was no major public awareness of biotechnology by stakeholders until Acceligen’s GnEd myostatin and polled cattle reached the point of concept discussion in 2013. Regulators—notably CTNBio in Brazil and CONABIA in Argentina—had designed and implemented case-by-case evaluation methodologies and reached regulatory landmarks for these cattle traits around 2018, and for GnEd Nile tilapia in 2019–2020. South American breeders and developers are keen to test the technology and evaluate its economic and commercial sustainability. The posture of activists and consumers in the region is largely unknown, however, and outreach to the general public, politicians, and social organizations would be timely.

## Summary of workshop breakout sessions

During two of the virtual workshop sessions, we held fourteen breakout group discussions with researchers, developers, regulators, and young professionals within regions (e.g., Europe and North America, Asia and Oceania, Latin America, and Africa) to discuss challenges and opportunities. Key points recognized across all groups included a need for improved communication by scientists and regulators to build public trust and science-based modernization of regulatory policies that do not prove restrictive to academic researchers and small developers.

### Communication

Livestock producers are tentatively interested in applications of GnEd that can improve production or efficiency but are concerned about uncertainties regarding the cost of bringing products to market and past public aversion to some technologies. For example, recombinant bovine somatotropin (rBST), a hormone injected into dairy cattle to increase milk production and approved by the FDA as safe in 1993 (U.S. Food and Drug Administration 1993, 2021), was rapidly adopted by much of the dairy industry and then subsequently abandoned due to consumer concerns. In this case, the public did not perceive sufficient benefits from the use of rBST to counter health and animal welfare concerns, and anti-biotechnology campaigns and misinformation were not effectively countered. Consumer acceptance of new biotechnology products clearly requires effective communication and consumer benefits, rather than only producer benefits as would be realized by some GM products.

Not all applications of animal biotechnology and GMOs are unpopular with consumers. For example, GM animals modified for pharmaceutical production have wide public acceptance in nearly all regions and have received regulatory approval in the European Union, the United States, and other countries (U.S. Food and Drug Administration 2009; Kling 2009; van Veen et al. 2012; Anonymous 2014; Shirley 2015). GloFish® offers several species and lines of fluorescent GM aquarium fishes that are popular aquarium pets in the United States and Canada (www.glofish.com), accounting for about 15% of all pet fish sales in the United States, although a recent poll (Pew Research Center 2018) found GloFish at the bottom of the list of GM animals supported by U.S. consumers. Further, plant-based meat substitutes created using GM yeast developed by Impossible Foods and other producers are increasingly popular with consumers who are vegetarians as well as those who normally eat meat but are concerned about environmental impacts and animal welfare. In the case of pharmaceuticals and meat substitutes, there are clearly communicated consumer benefits—life-saving drugs that are not easily produced by other methods and food sustainability. GM animals lacking a clear consumer benefit face broader resistance from consumers and environmental groups.

Communicating consumer benefits of animal biotechnology was recognized by all regional discussion groups as an area needing greater attention. As noted, scientists enjoy explaining the technical aspects of biotechnology, but do not always effectively convey societal benefits. Non-scientists are generally not interested in genetic mechanisms or increases in efficiency benefiting producers but are more receptive to outreach if their concerns are directly addressed. For example, a narrative about improved efficiency to reduce greenhouse gas emissions may be better received than one on production efficiency. Recommendations included training for scientists to engage in active societal dialogue, not just to teach the science. Training programs in science communication exist for students and are increasingly viewed as important. Many scientists are already highly effective communicators, yet are limited by time, resources, and funding. Public communication requires incentives, such as funding and institutional support. Finding the right balance between research and public outreach is an ongoing challenge.

There are several GM and GnEd animals that are being developed to specifically address consumer values and producer needs. Many animals that have been developed with the potential for commercialization have traits centered on animal and worker welfare, rather than traits solely for production costs, taste, or nutrition. For example, pigs modified to be resistant to porcine reproductive and respiratory syndrome will not suffer or be lost due to the disease. Dairy cattle modified to have the *polled* allele will have to not undergo the painful practice of disbudding. Comparatively, producing the hornless trait in dairy cattle using conventional breeding would take many generations, with significant losses in production. The environmental impact of meat production is gaining public awareness, particularly regarding greenhouse gas emissions. Public support might be gained by framing GnEd as a way to quickly reduce environmental impacts through improved efficiency in ways not possible with conventional breeding.

An example of a GM animal that meets consumer demand in a very specialized niche market and not focused on producer needs is the GalSafe pig, a GM pig engineered to eliminate the alpha-gal sugar on cell surfaces. This GM animal was developed for biomedical applications, such as xenotransplantation and porcine-derived drug products like heparin that are free of detectible alpha-gal. Given the rise in certain red-meat allergies, the company also sought approval for food use. While the U.S. Food and Drug Administration did not evaluate the allergenicity and food safety of this product for people with Alpha-gal syndrome (AGS), an allergy to red meat resulting from a Lone Star tick bite, since the product is free of detectible alpha-gal sugars, it should allow people with this allergy to safely consume this pork (U.S. Food and Drug Administration 2020). This GM pig, however, is quite restricted regarding rearing conditions and is not approved for production on conventional farms. Therefore, it fits into an unusual category of a GM trait that only serves to benefit consumers with AGS, without conveying any benefit to farmers.

How the distinction between GM vs GnEd products may affect consumer acceptance is not certain. Precise edits of single nucleotides or the addition of a trait from the same species, equivalent to what could be achieved through conventional breeding, may be more acceptable to consumers if clearly communicated. Genes inserted from other species will likely continue to face increased scrutiny. The regulatory approaches of some countries have tiered oversight, with different assessment requirements for these different changes. As noted above, some countries are taking different approaches to regulating GM and GnEd animals, which will both influence and be influenced by public perception.

### Regulatory approval

Regulatory approval is a long and uncertain process in many countries, and regulatory uncertainty is a significant concern to both developers and academic researchers. The experience of AquaBounty, with a 20-year regulatory and public acceptance pathway, has led to hesitancy among companies to invest heavily in animal biotechnology. Animal agricultural companies are awaiting examples of successful applications to pave the way for public acceptance. The regulatory landscape for animal biotechnology is continuing to evolve globally, with growing distinction between classical genetic modification using rDNA and more recent GnEd approaches used to generate changes that could occur through mutagenesis or conventional breeding.

Unnecessary costs associated with non-risk-based regulatory frameworks may limit developments. Regulatory frameworks in some countries are costly and difficult to navigate, and can prove prohibitive to innovation, especially for academics and small companies developing technology. Public policies directly affect the choice of research topics. For example, base-pair and gene deletions are easier to gain approval for through regulatory pathways than insertions, and thus research focuses on developing new traits through deletions. In Canada, proponents must put an economic value on a trait, which means that only high-value traits are targeted. In several countries, there are two or more agencies involved in the approval of a product. For example, in Australia food safety approval (conducted by FSANZ) is distinct from approval of the animal for commercial release (conducted by the Office of the Gene Technology Regulator). In Canada, it was recommended that a food safety evaluation be done for a GnEd animal although not required, as the trait was not novel, to “make the public more comfortable.” Of great concern to developers, breeders, and scientists are the lost opportunity costs—every year that existing technology is not used means avoidable costs in terms of disease prevention, animal welfare, and global food security (Van Eenennaam et al. 2021).

Public trust in regulatory approaches and decisions is critical. Regulations may enable public trust by reassuring the public that products are safe, but also may generate safety concerns unsubstantiated by science (i.e., “if this is safe, then why is it so strictly regulated?”). Likewise, how the product is regulated—e.g., as an animal drug or as a food animal —may alter consumer perception of safety in different ways. This may be particularly confusing in the United States because a single nucleotide deletion in plants is exempt from biotechnology regulation, yet the same type of edit in an animal is regulated as a drug. Further, perception of agricultural biotechnology is also closely tied to negative perceptions of large corporations. Ironically, as seen most clearly with the plant biotechnology sector,^1^ the costly regulatory process allows mainly large corporations to gain approval of their products and limits commercialization of products developed by the academic or public sectors and small businesses, as well as limiting the type of traits developed (Whelan et al.  2020). This situation likely compounds public mistrust of such corporations and increases mistrust of the products at issue. Developers suggested that regulations should be streamlined so that small firms and academic institutions also can afford the approval process and thereby shift GM and GnEd products from the association with large corporations. Overall, science-based regulatory development and modernization was recognized as a critical issue for moving animal biotechnology forward.

### Marketing and trade

As countries continue to develop regulations, sharing guidelines and experience is key to encouraging harmonization to enable international trade. Currently, variation among regulatory systems and regulatory uncertainty have limited international trade of animal biotechnology products in several regions. Moreover, differences in definitions make tracing products of biotechnology, when required, challenging and expensive. New products will likely have to be accepted on a market-by-market basis as regulatory approaches and frameworks develop. The challenge of regulatory approval has caused companies to avoid pursuing marketing in some regions, while at the same time still engaging with regions that have a large influence on global developments (e.g., the European Union). Often developers will delay the commercialization of new products until export is possible into major markets, as demonstrated in the seed industry, where seeds for new approved crops are not released until approvals in key markets are obtained, delaying farmer access to new traits. Mutual recognition of regulatory decisions would facilitate trade and encourage innovation. There is some alignment of regulatory processes among South American countries.

Labeling presents a particular challenge for trade of products from GnEd animals that do not have new DNA sequences or edits that distinguish them from conventionally-bred animals. The need for labels based on process when two products cannot be distinguished was questioned by researchers and developers. Already a lot of information that could be provided on labels is not (e.g., cattle breeds, antibiotic use), even when consumers may care about these characteristics. Part of the challenge is to avoid misleading consumers with labels when the meaning to the public is not clear. The objective of the label needs to be critically evaluated. In some cases, labeling products of GnEd could be beneficial if the product develops a reputation for sustainability or improved safety and may occur voluntarily, although this is far from certain.

Marketing and trade require some degree of agreement or mutual understanding relative to regulatory processes among trading partners. With GM products developed with rDNA insertion, the regulatory processes and timelines are not harmonized globally, resulting in asynchronous approvals. However, historically there has been general agreement among most countries regarding the definition of rDNA-derived plants and animals and what types of products are regulated. One major challenge with lack of harmonization of regulatory approaches for products of GnEd could be that different countries will make differing determinations as to the types of GnEd products that are regulated as conventional products versus those that will be regulated under their GMO regulations.

### Regional aspects

Much trade, research, and animal selection occur within regions. Because most livestock trade occurs within regions, we consider the development of animal biotechnology policy and related issues within selected regions.

African researchers using GnEd could provide solutions appropriate to regional needs. For example, non-adapted but highly productive cattle breeds could be edited to express the necessary drought, heat, and disease resistance traits required in Africa, to rapidly improve food security and reduce the environmental impact of cattle production. While these results could be achieved using conventional backcross breeding, the time required to achieve large-scale outcomes is unaffordable. Before GnEd can be employed, issues of training, funding, and access to technologies must be resolved. Moreover, African countries are challenged by fragmentation of capacity in the public sector and low acceptance of biotechnology. Currently, Kenya, Nigeria, and South Africa are most actively involved in developing regulatory approaches regarding products of GnEd.

With the exception of Japan (Tsuda et al. 2019), regulations for GM and GnEd animals in Asia are under development, pending, or do not exist. In general, Asian countries face similar challenges of public acceptance, lack of research and development support, and technical expertise. Many countries lack experience in safety assessments of GM animals, clarity regarding regulatory pathways, public confidence in food safety of GM products, and public understanding of benefits of GM products. However, there is also considerable variation in public support and engagement among countries. For example, the Philippines has good and positive engagement with the public and stakeholders, with high levels of trust for safety in regulatory authorities. Regulatory harmonization, especially in environmental risk assessments, may be difficult to achieve because of the complexity and differences in environmental conditions and public perception.

In Central and South America, Brazil and Argentina have well-defined regulatory pathways for biotechnology, and though regulatory policies for GnEd animals are under development, the two countries are poised to lead the world in adoption of products of animal biotechnology. Both countries have made regulatory determinations for GnEd products, deeming products that do not contain “foreign” DNA as conventional. These two countries serve as an example to low- and middle-income countries with limited regulatory resources that are developing animal biotechnology regulations and regulatory approaches for products of GnEd. An increasing number of Latin American countries are aligning with Argentina and Brazil.

The United States and the People’s Republic of China have large agricultural economies and both have invested heavily in development of agricultural biotechnology. Should they develop and implement policies enabling adoption of GnEd animals, due to their global economic impact, this would likely lead more countries to develop trade- and innovation-enabling regulatory policies.

## Conclusion

Many challenges remain for animal biotechnology to gain trust and acceptance and to become available for farmers to use to meet global demand for food products while improving animal welfare and reducing environmental impacts. Thoughtful communication can help consumers become aware of the benefits to themselves (e.g., food prices/availability, enhanced nutrition), animal welfare (e.g., reduction of diseases; cattle that will not need to be dehorned; pigs that will not need to be castrated), and the environment (improved efficiency reducing inputs and greenhouse gas emissions; aquaculture production reducing demand on natural fisheries and using fewer resources), as well as the costs of inaction (prevalence of preventable diseases, environmental costs). The degree of adoption of animal biotechnology will be determined by public perception and the development of regulatory approaches that are science-based and risk-proportionate. Many critics of animal agriculture and biotechnology emphasize the need to reduce or cease the use of animals due to environmental impacts and welfare concerns. These concerns are valid to some extent; however, demand for animal products will continue and will increase in some regions for the foreseeable future. Genome editing technology offers an opportunity to rapidly improve animal welfare and reduce environmental impacts that would otherwise be cost-prohibitive using conventional breeding and if not cost-prohibitive, could take decades to achieve.

The community of professionals that participated in the virtual workshops identified steps that might be taken to achieve the conditions needed for GM and GnEd animals to be used in practical applications in agriculture. Building human infrastructure is critical for effective regulatory oversight. Regions developing regulations need to train scientists on regulatory systems and communication, not only to support oversight of animal biotechnology and to create the next generation of regulators. Training is needed for assessing animal biotechnology, animal welfare, and food and environmental safety. Facilitating international dialogue and training is especially important in regions that are developing regulations. Continued communication between scientists, developers, breeders, farmers, regulators, and the public is necessary for all sides to build trust and transparency. Assisting journalists, teachers, and politicians in their understanding of the basics of biotechnology and value of its applications is essential. Workshops for training scientists in public communication, as well as regional and global workshops to disseminate information on research, regulations, and communication best practices, are ongoing or under development. Communication strategies must be designed for particular countries and regions based on the needs and concerns of the regional public. Regulatory alignment or compatibility and modernization of existing regulations are important to enable development and trade, while encouraging innovation. Regulatory alignment, at least within and among some regions, would reduce non-tariff barriers to trade that limit growth of the industry, particularly for smaller companies. Regional workshops and meetings will allow continuing information-sharing, especially of regulatory guidelines and case-studies of successful and unsuccessful applications.

## Data Availability

Supporting material may be found at: https://sites.google.com/a/vt.edu/animalbiotechresources/2020-online-workshops.

## Footnotes

1 There is only one GM animal developed for food use on the market; therefore, it is necessary to look to the plant biotechnology sector for examples and analysis of impacts of regulatory approaches.

## Glossary of terms

CRISPR-Cas9: Clustered regularly interspaced short palindromic repeats) is a family of DNA sequences found in the genomes of prokaryotic organisms; derived from DNA fragments of bacteriophages that had previously infected the prokaryote, they are used by the host to detect and destroy DNA from similar bacteriophages during subsequent infections. Cas9 (or "CRISPR-associated protein 9") is an enzyme that uses CRISPR sequences as a guide to recognize and cleave specific strands of DNA that are complementary to the CRISPR sequence. Cas9 enzymes together with CRISPR sequences form the basis of the CRISPR-Cas9 vector that can be used to edit genes within organisms (Doudna and Charpentier 2014)
Environmental risk assessment (ERA): Adjective, referring to an animal subject to classical gene transfer methodologies whereby an introduced recombinant DNA construct integrates randomly into the host genome, or to its descendants that inherit that construct
Genome edited (GnEd): Adjective, referring to an organism in which a nuclease (such as zinc finger nucleases, TALENs, or CRISPR/Cas9) creates a change (insertion, deletion, or substitution) at a targeted site in the host genome
Living Modified Organism (LMO): Any living organism that possesses a novel combination of genetic material obtained through the use of modern biotechnology (Secretariat of the Convention on Biological Diversity 2000)
Modern biotechnology: The application of: a. In vitro nucleic acid techniques, including recombinant deoxyribonucleic acid (DNA) and direct injection of nucleic acid into cells or organelles, or b. Fusion of cells beyond the taxonomic family, that overcome natural physiological reproductive or recombination barriers and that are not techniques used in traditional breeding and selection (Secretariat of the Convention on Biological Diversity, 2000)
Regulatory cooperation: The process whereby two countries or supranational entities work together to achieve a regulatory outcome in the absence of efforts to adopt similar policy structures
Regulatory harmonization: The process whereby two countries or supranational entities seek to establish similar regulatory policies
SDN1 genome editing: produces a double-stranded break in the genome of a target organism without the addition of foreign DNA. The spontaneous repair of this break can lead to a mutation or deletion, causing gene silencing, gene knock-out, or a change in the activity of a gene
TALENs: Transcription activator-like effector nucleases are restriction enzymes that can be engineered to cut specific sequences of DNA. They can be introduced into cells for use in genome editing
ZFN, zinc finger nucleases: Artificial restriction enzymes generated by fusing a zinc finger DNA-binding domain to a DNA-cleavage domain. Zinc finger domains can be engineered to target specific DNA sequences, enabling targeting of unique sequences within complex genomes. By taking advantage of the endogenous cellular DNA repair machinery, ZFNs can be used to precisely alter the genomes of an organism.
